# Overview about the keloid scars and the elaboration of a non–invasive, unconventional treatment

**Published:** 2010-05-25

**Authors:** I Carantino, IP Florescu, A Carantino

**Affiliations:** ‘Bagdasar–Arseni’ Clinical Emergency Hospital, BucharestRomania

**Keywords:** pathologic scars

## Abstract

Although the healing means 2 types of biological mechanisms that seem to be ‘pathologic’, the swell and the granulations are a normal process in the biology of the human being, representing two systemic functions: the adaptation and the morphogenesis. There is a pathological healing in which the fundamental healing phenomenon is deviated from the normal. There are three variable parameters responsible for the pathological evolution of a scar: the cellular population, the fundamental matrix and the fibers. The healing evolution can be deviated to an intense maturation with an ‘old’, atrophic scar, or to an incomplete maturation and the result is a hypertrophic or a keloid scar. For the hypertrophic scars, the excision and the skin graft lead to good results and the relapses are rare; the keloid relapse is always at the border between the graft and the wound edge, or between the two skin grafts. These are the considerations for which the treatments are mixed, combined (surgical, drugs, physiotherapy) both in our country and abroad, but the results are still frustrating. That is why new, modern methods of treatment are used today: criotherapy, laser, ultrasounds. However, even those treatments are not very successful: tissue expander, external press therapy, corticosteroids injections, other pharmacological agents (retinoic acid, colchicines, antineoplasics). We propose a regenerative, alternative, non–invasive treatment starting from the results we obtained in a research work 4 years ago, when we irradiated the fibroblasts in an electromagnetic high frequency millimeter waves field, and we obtained the fibroblasts apoptosis and the reorganization of the collagen fibers by changing the piezoelectric emission

The only sure healing, without future problems, is the **regeneration = the replacement of a certain tissue with the same type of tissue**, without any dysfunctions.

The cicatrization requires the stopping of the lesion with another kind of fibrous tissue, representing a ߢpermanent weak point’ of the respective structure. It means that some requirements of the local mesenchymal population, of the epitheliums, inflammatory changes, aggressive blood cell migrations, increasing quantities of enzymes, cellular mediators and modulators, need to be complied. 

Although the healing means 2 types of biological mechanisms that seem to be ‘pathologic’, the swell and the granulations are a normal process in the biology of the human being, representing two systemic functions: the adaptation and the morphogenesis. There is a pathological healing in which the fundamental healing phenomenon is deviated from the normal. There are three variable parameters responsible for the pathological evolution of a scar: the cellular population, the fundamental matrix, and the fibers. The pathological evolution is produced by:

- deviations in the continuity of the healing
- deviations in the reactivity of the organism
- deviations produced by the traumatic agent

**Figure 1:**
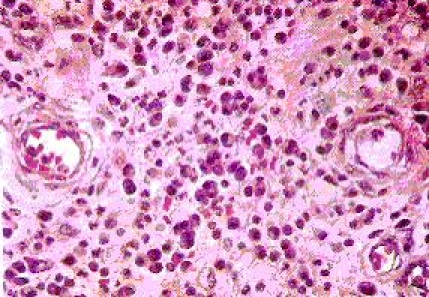
Granulation tissue with inflammatory cells and congested blood vessels; hematoxylin–eosin color (objective 40x)

**Figure 2:**
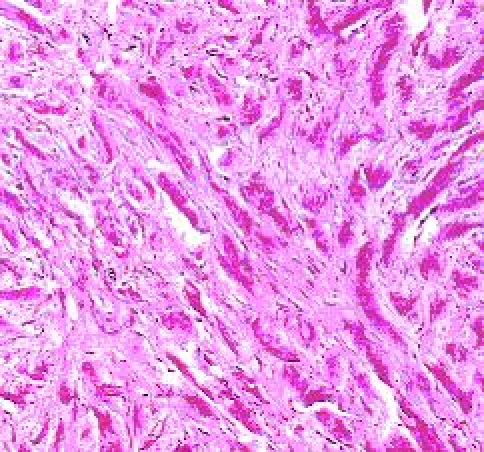
Keloid with abnormally large collagen fibers and large fibroblasts; hematoxylin–eosin color (objective 40x).The healing evolution can be deviated to an intense maturation with an ‘old’, atrophic scar, or to an incomplete maturation and the result is a hypertrophic or a keloid scar.

**Figure 3:**
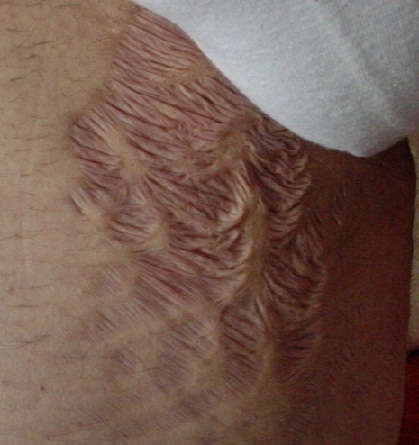
Atrophic scar in a 28–year–old man, after losing 20 kg

**Figure 4:**
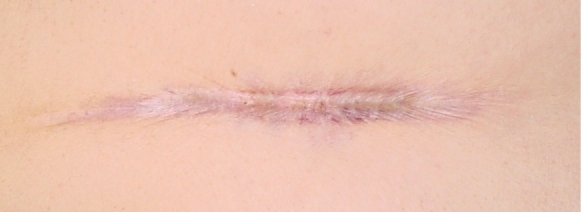
Retractile scar after a hypertrophic scar, in a 19–year–old girl, operated at 4 years old

**Figure 5:**
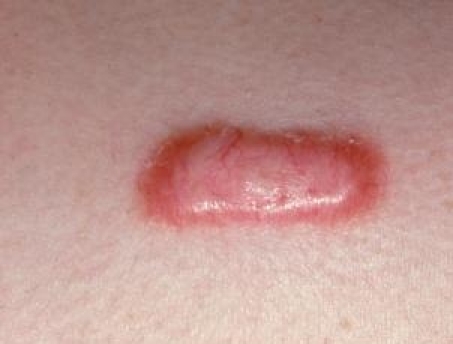
Keloid scar after acneea vulgaris

There are 2 modeling steps of a scar: a precocious immature step with swelling accesses and local accumulation of fundamental matrix, and a late, mature step with vascular and cellular involution and increased local fibrillar density. The hypertrophic scar and its tumor form = keloid, develop after a **chronicle stagnation process in the modeling phase from the immature period**, either by the persistence and continuous self reproduction of big amounts of ‘imperfect’ metaplasia products and/or extracellular structures, or from a great irritability (or from an increased production of cytokines and TNF) of T–Lymphocytes responsible with the recognition and tolerance of the new structures. 

In the first mechanism (imperfect structures inflation) the hypertrophic scar develops. 

In the second mechanism (strong vigilance of the lymphocytes), the result is the keloid [1]. 

Biochemical investigations demonstrated a great increase of the active fibroblasts synthesis in the keloid, followed in order by the hypertrophic scar, the normal scar and the normal skin. The proteinglicans level goes parallel with the collagen synthesis. It was observed that this cellular activity could be reproduced in the cells cultures by treating the fibroblasts with growth factors due to the modern research possibilities in the cellular modulators and mediator's field [2, 3, 4]. Some authors (Badalamente and contributors, 1992; Kolen and contributors, 1995), using imunohistochemistry tests, observed an increased activity and an increased level of growth factors (**PDGF** (platelet derived growth factor), **EGF** (epithelial growth factor), **TGF β **(transforming growth factorβ) [5].

According to their evolution, the scars are [1]:

- hypertrophic = with a short evolution
- cheloid = with a long evolution
- heteromorphic = with an intermediary evolution

The hypertrophic scars develop after a wound; the scar is red, irritable, with a high throwing into relief, with cyanosis areas and pruritus in the swelling accesses; they whiten at pressure and grow quickly (3–6 months) and after that, the scars partially regress (scar with a short evolution). It appears everywhere on the human body and does not invade the normal skin; the frequency is bigger at the age of 5–7. The scars with a great volume cannot regress (even after the signs of activity disappear) because there is a big amount of collagen in them. 

The keloid scars have a long evolution (years), are growing all the time and invade the normal tissues around. The keloids look like tumors, are warm, red, firm, itching, with exfoliations. These forms are often on the back, breastbone, hips; thighs (they are rarely found on the penis, eyelids, areolas, scrotum). Keloids are found only in humans and occur in 5–15% of the wounds. These scars develop in young adults and, usually, not before puberty, never on the palms and soles; the average age at onset is of 10–30 years old; they are more often affecting the Africans, where the transmission is autosomally dominant. The evolution is permanent, progressive and uninterrupted. 

Keloids and hypertrophic scars are genetically associated with HLA–B14, HLA–B21, HLA–Bw16, HLA–Bw35, HLA–DR5, HLA–DQw3, and blood group A [6].

For a certain diagnosis between the hypertrophic and the keloid scar, one has to examine the limits of the scar in the normal tissue around, if the keloid infiltrates, invades, and penetrates the normal tissue.

After Muir [1, 7, 8]:

- something is missing in the normal derma near the keloid, which usually resists the fibroblast invasion in the normal or hypertrophic scar;
- the fibroblasts from the keloid scar represent a different population compared to the normal, natural populations.

Calman and Copenhagen [9] transplanted cheloid from the breastbone to the abdominal skin; the cheloid regressed while the donor site scar became cheloid even if it had been operated with skin graft from the abdomen. This test shows that the cheloid scar development is not controlled by abnormalities of the local fibroblasts, but there is a deficiency of a defending factor of the skin (mechanical or biochemical) [1, 7].

Russel and contributors (1988) demonstrated that the fibroblasts from the cheloids need less stimulation as far as the growing factors are concerned in order to proliferate in the cultures than the normal cells. Their conclusions were that the cheloid–cells are less exact than the normal cells and that they develop in a great density in the mediums with less growing factors [1, 7].

**The collagen fibrils are abundant, immature and unorganized** (they are more irregular, abnormally thick, and have unidirectional fibers arranged in a highly stressed orientation). 

**Figure 6:**
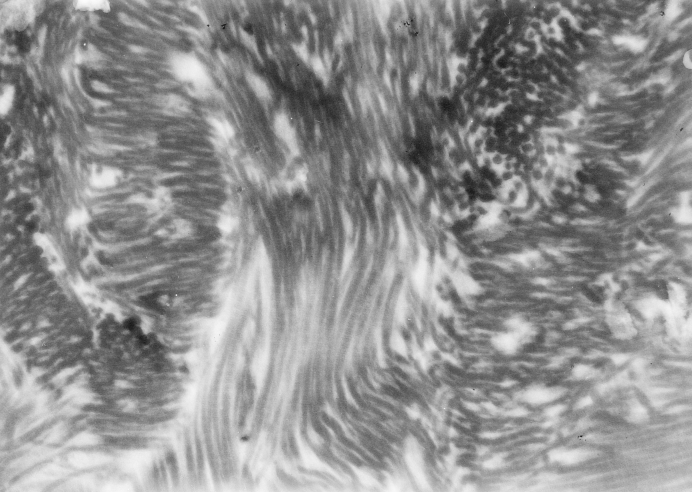
Fascicles of collagen fibers (keloid scar); Philips 206 S electron microscope

The epidermis layer has a quasi–normal aspect. We remark a diffuse fibroblast–histocytes proliferation and some rarely disposed chronically inflammatory elements at the dermis level. Moreover, pericellular configuration appears like a collagen tissue and the proliferation zone extends near the hypodermis layer (Figure 7)

**Figure 7:**
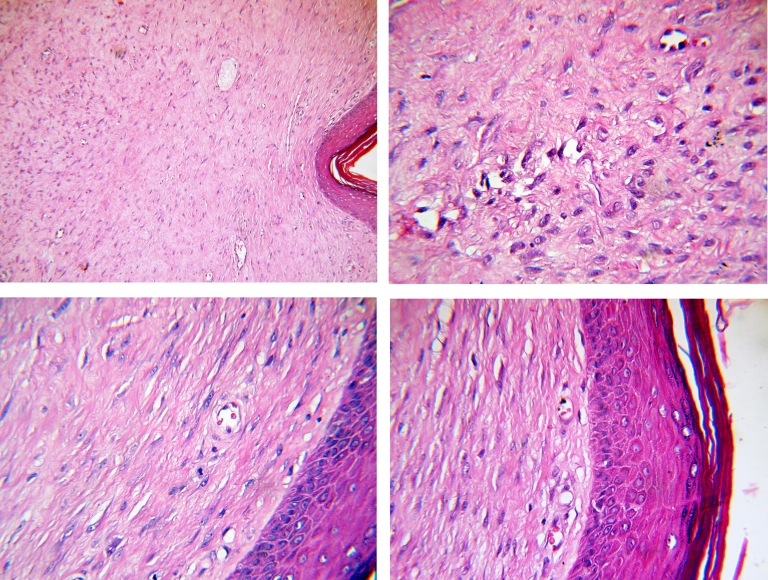
Keloid scar–hematoxylin–eosin color, optical microscopy [Novex Holland optical microscope, Carfem Electronic Ltd]

**There is equilibrium between the collagen synthesis and the destruction in the normal mature scar.** In the keloids' case, the immaturity is explained by a failure of the equilibrium favorable for the synthesis, the collagen destruction being normal. We can see the immaturity at many levels (collagen, fibroblasts, glycozaminoglicans, miofibroblasts, enzymatic and immunity markers, mastocytes). The collagen is structural and histochemically immature. Type 3 collagen is more immature in comparison with type 1 collagen (normal, mature collagen). **The fibroblast proliferates and the metabolic answer is greater (instead of diminishing).** Miofibroblasts are responsible for the keloid contracture. Mastocytes are normal in the normal scar after maturation and, are more in the keloid, being responsible for the congestive accesses. The arterial supply is abundant and is responsible for the red color that becomes pale after pressure and with the cyanosis areas during the swelling accesses. This intense arterial supply with embryonic characters keeps the anti–tissue aggressiveness of T lymphocytes. The enzymatic changes are the increase of the energetic metabolism enzymes, the decrease of the proteolitic enzymes, with the increase of the protein synthesis. The increase of the acid phosphatase and glyco–6–phosphate–dehidrogenasis is a characteristic way of evolution for keloid scars. From the immune point of view, the local increase of IgG, α1 antitrypsin and 2 macroglobulin is compulsory.

The modern biology and physiology tissue studies brought out the concept of epithelial–mesenchymal transition [7, 10, 11]. The epithelium and the mesenchyma represent the two fundamental cellular phenotypes of the organisms. These two phenotypes have different intracellular structures and different extracellular bundles with other cells and with the extracellular matrix. However, it was demonstrated that these different cellular types are interconvertible. The first mechanism of epithelial–mesenchymal translation consists in the molecular effect of adhesion between cells. The second mechanism is that of integrins, which assure the connection between the mesenchymal cells and the intercellular matrix. The third mechanism consists in the effect of the ligand molecules on the cellular receptors, which (after becoming activated) initiate ways of signalizing, conducting to cellular reorganization. These particular mechanisms of the normal cells and tissues can be adulterated by different pathological situations. Changes of the intern medium, the composition or structure of integrins, adhesins, cytokins produced by fibroblasts [1] and mostly by the vascular ones [7] are responsible with the cellular changes including differentiation, epithelial–mesenchymal translation and even malignity (Marjolin ulceration). Qualitative and quantitative changes of tenascyna, fibronectines, glucosaminoglicans, the adulteration of the fibronexus (the matrix and the intracellular fibers αactin, desmin, vymentin, microfibers and microtubulatures), were found. The changes in the structure and chemistry of the ligand cells are produced by the oncogenes or by the alteration of the enzymology in the tissue medium. 

The phenotypical translation from epithelium to connective and reverse represents an important signal for the beginning of the healing phenomenon. The transformation from epithelium to mesenchyma and reverse is called metaplasyc transmutation. The ration **cell–fundamental matrix** is controlled by the blood vessel that secreted the cytokines, quinines, prostaglandines, eicosanoides [1]. 

For the hypertrophic scars, the excision and the skin graft lead to good results and the relapses are rare; the keloid relapse always at the border between the graft and the wound edge, or between two skin grafts. 

## Fetus cicatrization

The fetus cicatrization starts in the same way the adult does, with intra and extra vascular coagulation, with an increase in the permeability of the vessels and the opening of the interstices, with the development of the fibrin barrier at the edge of the hotbed and a massive healing granulation.

What is missing, in comparison with the adult cicatrization is the action of the specialized cells for phagocytosis, which are responsible for the pain and reactivity of the adult's wounds.

The fetus and embryo cells contain, just like the adult's cells, the enzymatic device responsible for the cellular suicide dissolution and disappearance; but these are centrally commanded, directly by the activity of the genes, and the commanding centre is always situated in the interior of the cell. In that way, there is no need for specialized killer cells, the embryo cells being able to sacrifice, an action that is often observed in the fetus–embryo enlightening.

What is specific for these cells, are the intense cytokinic activity, conducting existing functions and becoming the same nature partners in the epithelium/mesenchym or endothelium/mesenchym ratio.

The absence of the important cellular aggressions, which produce the adult's tissue shortcomings and permanent cytokinic activity, fundamental for the life of these cells, can explain the healing without scar, by means of the tissue regeneration.

After Kratz and contributors (1993), the healing of the fetus wounds inside the pregnant uterus is fundamental, different in the adult, in accordance with the result of the healing, and according to the way of healing, this being due to the amniotic liquid (Samasundaram and Pratham, 1972). The last authors demonstrated that the deviation of the amniotic liquid from the uterine cavity, disturbs the fetus cicatrization (Dahl and contributors, 1983; Kujawa and Tepperman, 1983) and Lomcarer and contributors (1990) demonstrated an important quantity of hyaluronic acid and amniotic liquid.

Moreover, Ackle (1981) indicates that the amniotic epithelial cells do not contain antigens (HLA–A–B–C–DR), and when these are subcutaneously implanted to volunteers, they do not induce rejection. The serum obtained from the volunteer hosts does not contain HLA anticorps. By using 3H Minida on cultures of human tissues (fibroblasts and keratinocytes) Kratz and contributors indicated that the amniotic liquid has mitogen effects like the supra insemination of amniotic cells. They suggest that the growth factors produced by the amniotic cells are responsible for the way of healing of the fetus.

The exocrine activity of the amniotic cells can explain (Adjick and contributors, 1985), at least partially, the influence on the healing of the wound, observed even in adult, while using the amniotic membrane as a biologic dressing (Colocho and contributors, 1974; Faulk and contributors, 1980). One can conclude that, the keratinocytes and fibroblasts of the fetus, are intensively stimulated by the amniotic liquid, at least in the tissue cultures. Green and contributors, Mulvihill and contributors (1979, 1986), Hagerstramd and contributors (1979), Talmi and contributors (1990), Kratz and contributors (1993), recommend (using these information), **the use of fresh, alive amnios grafts and the application of the amniotic liquid in the prophylaxis of hypertrophic and keloid scars.**

Laboratory data (Krummer and contributors, 1988) show that the fetal scar can become hypertrophic by treating the wound with TGF–beta, suggesting that the healing of the fetus and embryo is not influenced by the cytokines.

Generally, there are some intrinsic and extrinsic differences between the fetus and the adult, probably influencing the healing (Garnier–Lyounet and contributors, 1993): the fetal skin is in a permanent warm, sterile, humid, hypoxic and rich in growth factors, ambiance. The amniotic liquid is a source of hyaluronic acid, fibronectine, and contains (Longaker and contributors, 1991) an inhibiting factor of the contraction, which is acting in a ratio with the dose.

## The actual stage of the clinical treatment of the pathologic scars

For the hypertrophic scars, the surgical excision and the grafts can induce good results with rare relapses.

The cheloid always relapses at the junction between the graft and the skin edge or between grafts or at the new suture level. 

These are the reasons why attempts of combined treatments (surgery, drugs, Rx–therapy, physiotherapy) have been made both in our country and abroad, but the results are only partially satisfactory.

Studies that lead to the development of the therapies associated with the surgical treatment of the pathologic scars have been made; however, they are more or less efficient (if we are speaking about costs and side effects). 

The tissue expanders lead to good results by removing the pathologic skin and replacing it with a healthy one, but it is a method which cannot be used everywhere on the surface of the body because there are some clear limitations (irradiated tissue, infections, chemotherapy, less vascularized tissues, psychological diseases).

Another method was the external pressotherapy by using clothes (gloves, pants, stays, cowls) or elastic dressings and silicon devices. This method is partially efficient as an adjuvant treatment.

The treatment of pathologic scars was always a touchstone for surgeons and that is why many pharmacological methods have been tried all over the world. Corticosteroids were directly injected in the little scars and together with the pressotherapy some good results have been obtained. Other pharmacologic agents used, were: colchicines (they decreased the fibroblastic secretion of the collagen, increased the collagenasic activity and inhibited the wound contraction in the systemic therapy); penicillamine, zinc salts (local applications), antineoplasics (were used for the tumor origin theory about the keloid scars, but there are too many risks of the treatment).

Retinoic acid (acts like a monokine with intense activity against fibroblastic secretion and proliferation being the strongest drug against keloid scars, but the costs are too high, and this is a limit for its use on a large scale): decreases normal tonofilament and keratohyalin synthesis, increases the production of mucoid substances and the epidermal cell growth rate, and inhibits DNA synthesis in vitro [12, 13].

Interferon (IFN) therapy showed a significant increase in the rate of scar improvement compared with the control period of time (P = .004) after injecting 9 patients who had hypertrophic scars, with 1 X 10^6 ^units of human recombinant IFN alfa–2b subcutaneously, daily for 7 days, and then 2 X 10^6^ units 3 times per week for 24 weeks in total. Scar assessment (P <.05) and scar volume (P >.05) also improved after 3 months of treatment. No recurrences were reported after stopping the IFN therapy [14].

## Our proposal for the development of a regenerative, alternative, non–invasive treatment of the keloid scars

5 years ago, we have started a research about the activated fibroblast with an increased installment of collagen synthesis (immature collagen type Ⅲ), after the irradiation in electromagnetic field produced by the millimeter waves (of extremely high frequency). The experimental investigations represented the theoretical base of the paper *‘Study of the irradiation effects in electromagnetic field produced by the millimeter waves upon cells of adulterated palmary aponeurosis in Dupuytren's disease’*. The most important observations are the following: the EHF–EMF irradiations produced an inhibiting effect on the activated fibroblasts in Dupuytren Disease, leading to the apoptosis of fibroblasts involved in the anarchical synthesis of collagen, and a new reorganization of the collagen fibers by changing the piezoelectric emission [15, 16]. 

A long time ago, it had been demonstrated (Cohen and contributors, 1971; Craig and contributors, 1975; Diegel and contributors, 1979) [6] that there are activated fibroblasts in the keloid, which present an increased installment, like in Dupuytren Disease.

If we take into account the experimental aspects discussed earlier and, corroborate them with the scientific background in the literature, we would be able to see the possibility that, in the initial stages of the scars development, this kind of unconventional treatment can lead the cicatrization biological process to normal healing, by preventing the deviation to pathologic healing. If the pathologic scars were already constituted, and if we associate the classical surgical treatment, it is possible to decrease or eliminate the relapse potential.
